# Efficacy of Nano Silver Fluoride and/or Diode Laser In Enhancing Enamel Anticariogenicity around orthodontic brackets

**DOI:** 10.1038/s41405-023-00151-x

**Published:** 2023-06-23

**Authors:** Aya Anwar Alsherif, Mohamed Ali Farag, Mai Badreldin Helal

**Affiliations:** Faculty of Dentistry, Tanta University, El-Giesh St., Tanta, Gharbia Egypt

**Keywords:** Health care, Diseases

## Abstract

**Purpose:**

This in vitro study aimed to compare the anticariogenic effect of using diode laser irradiation and/or nano silver fluoride varnish around orthodontic brackets.

**Materials and methods:**

60 caries-free and intact premolars were randomly divided into 3 experimental groups as follow: (1) Group I (nano silver fluoride treated group, *n* = 20), (2) Group II (diode laser treated group, *n* = 20) and (3) Group III (combined nano silver fluoride and diode laser treated group, *n* = 20). Anticariogenicity was assessed using polarized light, scanning electron microscope, elemental and shear bond strength analyses.

**Results:**

PLM and SEM showed presence of few demineralized areas in group I. Group II revealed a dramatic increased demineralization. Group III disclosed almost typical homogenous surface enamel. elemental analysis showed a highly significant difference between Group III and II and a significant difference between Group III and I. Shear bond strength analysis revealed a significant difference between group I and II and between group III and II. The difference between group III and I was non-significant.

**Conclusion:**

Both diode laser and nano silver fluoride positively affected dental enamel with the most superior enhancement in enamel criteria was achieved by surface pretreatment by combined nano silver fluoride varnish and diode laser irradiation.

## Introduction

Dental decay in the form of white spot lesion is a very common undesirable side effect during orthodontic treatment [1]. The use of either fixed or removable orthodontic appliances on tooth generate non-cleanable surfaces causing dental plaque accumulation, alteration of oral flora and therefore they result in areas of initial enamel decalcification [2, 3]. Histologically, initial enamel decalcification or/ white spot lesion is defined as a sub-surface enamel porosity created by partial dissolution of enamel crystals [4]. Enamel is said to be a precious tissue as once it is lost, it can’t be restored. This is because that its forming cells, ameloblasts, are lost with tooth eruption unlike other hard tissues whose cells remain throughout life time. Accordingly, the demineralization occurring in early enamel lesions is seen to be a reversible process under favorable conditions. However, more advanced demineralization is essentially irreversible. various interventions, including chemical and biological approaches, were used to restore newly formed enamel lesions before they develop into actual surface cavitations [5]. Considering the chemical approach, several in vitro studies analyzed the ability of many remineralizing agents to remineralize white spot lesions around orthodontic devices. For example, topical fluoride application [6], fluoride releasing adhesives [7] and casein phosphopeptides amorphous calcium phosphate (CPP-ACP) application [8].

Recently, researchers portrayed that enamel surface treatment with specific anticariogenic agents, prior to orthodontic management, is very helpful in preventing white spot lesions from being created. An emerging technique, including the use laser irradiation with or without incorporating a remineralizing agent, is being studied lately [9]. This technique was proved to constrain enamel surface demineralization during orthodontic treatment via creation of microstructural changes in enamel, leading to a marked increase in enamel acid-resistance ability [10]. For example, the use of argon laser during orthodontic appliance cementation was proved to reinforce the enamel to acid attacks and reduce the possibility of enamel surface demineralization around orthodontic brackets [11]. Similarly, many in vitro studies achieved a marked acid-resistance ability of enamel using CO2 laser [12], (Er: YSGG) [13, 14] and diode laser application [15, 16].

Depending on the fact that nano-sized particles have resemblance to the apatite crystals of tooth enamel in morphology and crystal structure, numerous studies established that using fluoride nanoparticles is significantly superior to usual fluoride in preventing dental caries [17]. On other hand, silver nanoparticles were also stated to be effective in caries prevention [18–20], thanks to silver nanoparticles innovate antimicrobial and cariostatic properties that were proved to work against dental decay pathogens [21]. Remarkably, an emerging regime, to create combined nano silver fluoride particles, proved to be effective in remineralizing early enamel caries and arresting dentinal caries [22].

Therefore, in our in vitro study, we proposed that combining laser therapy with nano-silver fluoride particles would be an exclusive formula to prevent dental caries. So, the effect of combined use of diode laser irradiation with nano silver fluoride varnish (NSF) on enhancing enamel anticariogenic activity around orthodontic brackets was evaluated and compared to the use of NSF or diode irradiation solely. Also, brackets bond strength to enamel were evaluated after treatment.

## Materials and methods

### Study design and sample assignment

This study was conducted as in vitro experimental study that was designed in accordance with the guidelines of the scientific research ethics recommendation of Ethical Committee at Faculty of Dentistry, Tanta University, Egypt (ethical approval #R-OB-9-22-5). An informed consent was signed by all subjects enrolled in our experiment. They were informed about our research protocol and how their samples will be used for scientific purposes.

A sample of sixty extracted human permanent maxillary first premolars was chosen according to certain inclusion and exclusion criteria. The inclusion criteria were: Sound fully developed maxillary first premolars that are usually extracted for orthodontic purposes. The exclusion criteria were: teeth showing any defects as microcracks, erosions, caries, restorations, or any visible defects buccally. After extraction, the teeth were stored in saline solution at 4 °C which was changed every day until the experiment was done. The sample size and power analysis were calculated using Epi-Info software statistical package created by World Health organization and center for Disease Control and Prevention, Atlanta, Georgia, USA version 2002. The criteria used for sample size calculation were as follows: 95% confidence limit and 88% power.

The teeth were randomly distributed using a computer-generated list of random numbers to one of three experimental groups as follow: (1) Group I (NSF-treated group, *n* = 20), (2) Group II (diode laser treated group, *n* = 20) and (3) Group III (combined NSF and diode laser treated group, *n* = 20).

### Sample preparation and subgrouping

**Fig. 1:**
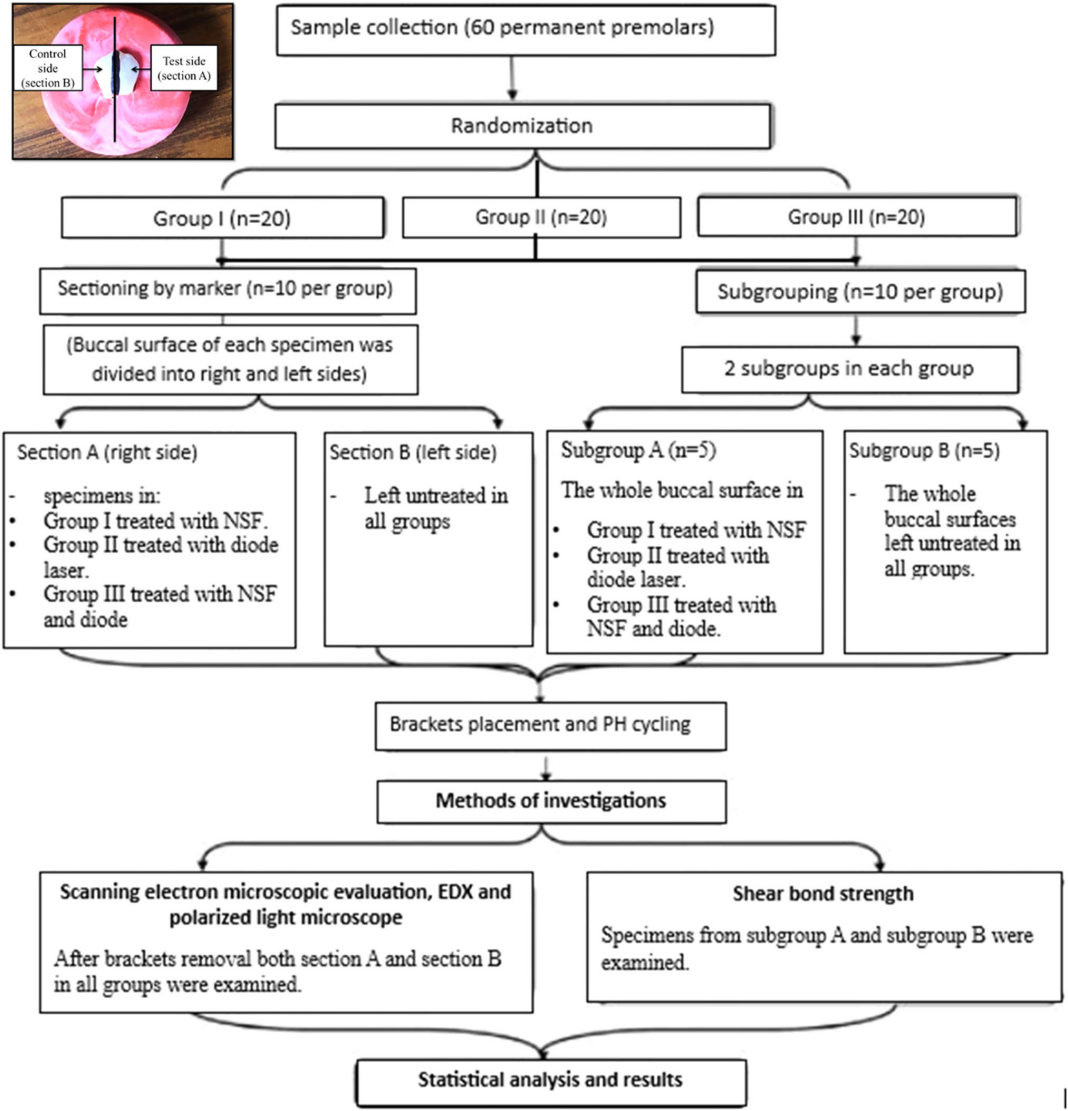
Flow chart illustrating the study design. (Inset) Photomicrograph showing sectioning of the buccal surface into right side (test side) and left side (control side).

First, all specimens were cleaned with fluoride free pumice then washed thoroughly with distilled water and air-dried. Afterward, for subgrouping purpose, the buccal surfaces of ten specimens from each group were equally divided longitudinally into two halves using permanent marker. In these specimens, the right half was set as a test side where enamel surface would be treated with its corresponding anticariogenic agent (named as section A). The left half was set as a control side where no treatment was applied (named as section B). The remaining ten specimens of each group were subdivided into subgroup A, where the whole enamel surface would be treated with its corresponding anticariogenic agent, and subgroup B where no treatment application was applied. These ten specimens were incorporated in shear bond strength measurement. Study design was shown in (Fig. 1).

- ***Experimental procedures***
  - **Nano silver fluoride application**
    The enamel surface of group I section A and subgroup A was treated with NSF varnish (Nano Gate Company, Cairo, Egypt). The varnish coating was applied by the aids of composite bond brush, left on the surface for 4 h then washed using distilled water as recommended by the manufacturer.
  - **Diode laser application**
    The enamel surface of group II section A and subgroup A was treated with diode laser. Laser irradiation was carried out using a 300 µm optic fiber conductor with a pulse diode laser at 980 nm wave length (Hu laser, K2 mobile, South Korea), a 0.8 W output power and an energy density of 5.33 J/mm2. The optic fiber oriented perpendicularly to the enamel surface with its tip held in contact mode for 30 s [23].
  - **Combined NSF and diode laser application**
    The enamel surface of group III section A and subgroup A was subjected to both NSF and diode laser conditioning. First, NSF varnish coating was applied followed by immediate laser irradiation to the enamel surface as previously described in Group II subgroup A. The specimens were then left for 4 h then the varnish was washed using distilled water.
  - **Bracket placement**
    After treatment application, premolar brackets (American orthodontics/ mini master series) with a base surface area of 10.25 mm^2^ were used for this study. bracket bonding procedure was followed in all specimens. First, acid etching was done using 37% phosphoric acid gel for about 15–20 s (Han ETCH37, HAN DAE chemical Co., LTD, Korea), rinsed with water then dried with compressed air. A thin coating of bonding agent (Rely. a. Bond, Primer) was applied with a microbrush on the treated enamel surfaces. Afterwards, No-mix orthodontic bonding material (Rely. a. bond, Adhesive Paste) was applied to the coated bracket base. The brackets were positioned on the facial surface at the height of contour mesio-distally, in the middle one third occluso-gingivally, and parallel to the long axis of the tooth. Finally, excess bonding material around the bracket was removed using a bracket placer sickle and light-cured for 45 s.
  - **PH cycling**
    Over a period of 14 days, all specimens of all groups were subjected to remineralization/demineralization pH cycles with a daily schedule of 6 cycles, where specimens were successively subjected to a demineralizing (120 min), a rinsing (30 s), a remineralizing (60 min), and again a rinsing (30 s) phase. During a 6 h “night” period, the specimens were exposed to the remineralizing solution. The composition of the remineralizing solutions was 1.5 m M CaCl 2, 0.9 m M KH 2 PO 4, and 20 m M HEPES with pH 7.0. The demineralizing solutions composed of 50 m M acetic acid, 2.2 m M CaCl2-H 2 O, 2.2 m M KH2PO4, 10 m M KOH, and 23.8 μ M NaF with pH 5.0 [24].

### Polarized light microscopic examination (PLM)

From each group, five specimens, which were divided into sections A and B, were included in this analysis. After brackets debonding, each tooth was equally sliced longitudinally in a buccolingual direction using low speed hand piece with a diamond disc to separate section A from section B. Now, each half could be evaluated independently as a ground section under PLM (Olympus America Inc.). For making ground sections, hand grinding method was used to get a 15 μm thickness buccolingual section of the specimens. Finally, sections were imbibed in water then observed under PLM to qualitatively evaluate the test area [23, 25]. The images were captured using light microscope built in camera (LEICA ICC50 HD Camera system) via image software LAS EZ version 3.0.0.

### Scanning electron microscopic analysis

From each group, five specimens, which were divided into sections A and B, were examined using SEM. After brackets removal, the specimens were thoroughly dried and gold-plated using JFC-1100E-IEOL ion sputtering evaporator. The specimens were then mounted on SEM (JSM-IT200, JEOL) for analyzing enamel surface morphology. In addition, the calcium and phosphate content of surface enamel was measured for each specimen on both sections A and B. This was achieved with the aid of SEM fitted with energy dispersive X-ray spectroscopy [EDX].

### Shear bond strength measurement

Ten specimens from each group, 5 specimens represented subgroup A and the other 5 represented subgroup B, were incorporated in this test. Each of the specimens (a tooth with an orthodontic bracket) was placed in a polyethylene container using DuracrylTM Plus self-polymerizing denture base resin (Spofa Dental, Jicˇín, Czech Republic). Each sample was dipped in such a way that the punch could be placed at the front side, tangentially to the tooth surface. To determine the adhesion value of an orthodontic bracket to the enamel, a shear bond strength test was performed using MTS 858 MiniBionix® machine (MTS System, Eden Prairie, MN, USA). A shear force was applied at the bracket-tooth interface until the bracket was detached, using the universal testing machine. The force required to take off the bracket was measured in Newtons (N) at a crosshead speed of 1.0 mm/minute. The SBS was then calculated in MegaPascal, as1MPa = 1 N/mm^2^, by dividing the force values by the bracket base area (10.25 mm^2^) [26].

### Statistical analysis

The quantitative data of EDX and shear bond measurements were collected, tabulated and statistically analyzed using CO-STAT analysis (version 6.4). Numerical variables were expressed by descriptive statistics as mean, standard deviation and range. One-way ANOVA and post hock test (tukey-test) were used to compare quantitative data between groups while independent *t*-test was used to compare between subgroups and sections in each group.

## Results

### Polarized light microscopic results

**Fig. 2:**
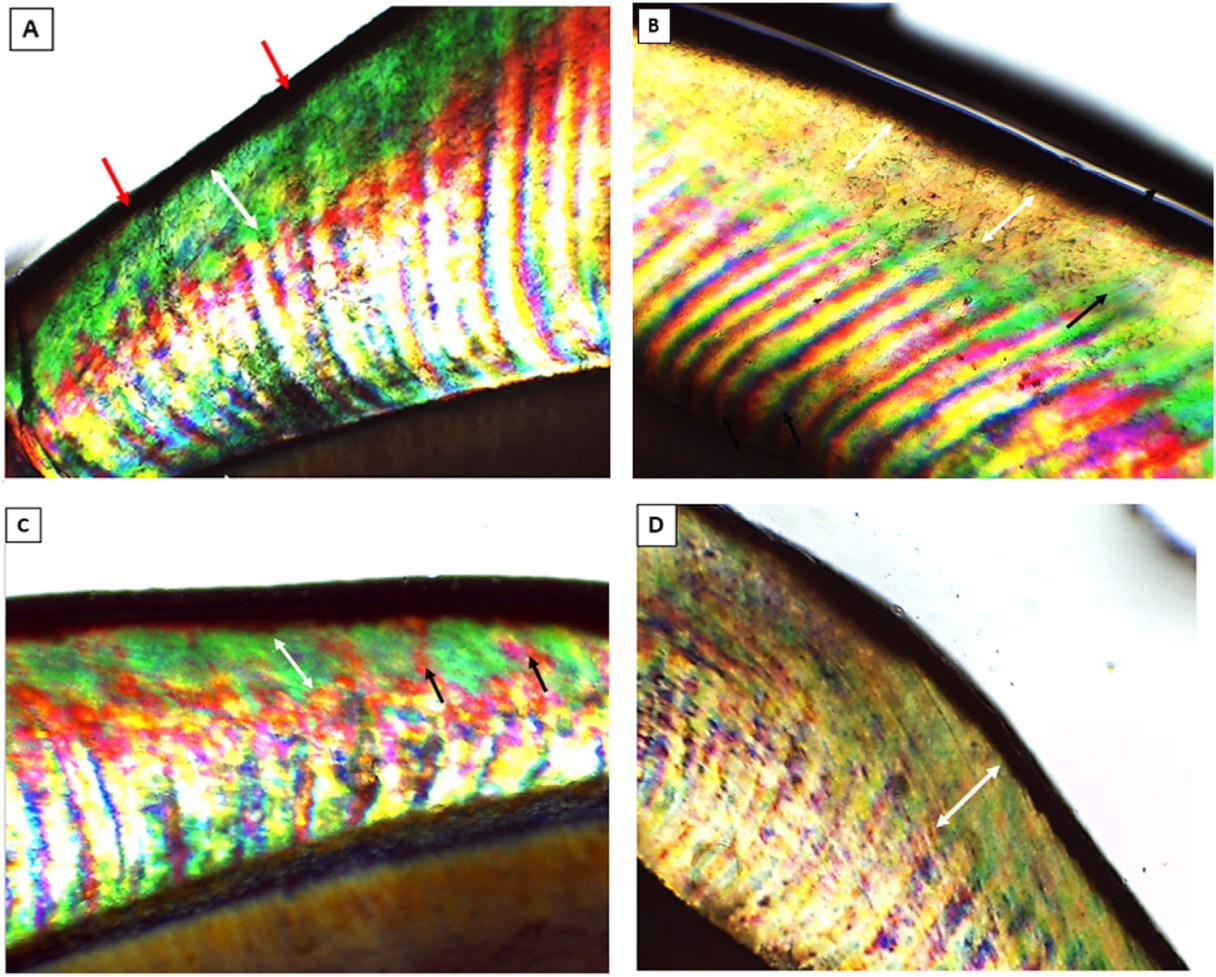
PLM of group I-B, group I-A, group II-A and group III-A. Polarized light photomicrographs of longitudinal ground sections showing, (**A**): group I section-B displaying a strong positive birefringent demineralized enamel band (*double head arrow*) extending beneath an intact surface layer (red arrows). **B** Group I section-A showing normal subsurface enamel (*double head arrow*) with few demineralized areas (*black arrow*). **C** Group II section-A exhibiting a widely distributed demineralized areas (*double arrow head*), though, still displaying minimal areas of remineralized enamel (*black arrows*). **D** Group III section-A showing almost typical homogenous subsurface enamel (*double head arrow*), reflecting normal mineralization and birefringence of enamel. (PLM, original magnification; A, B, C, D × 40).

After PH cycling, the test areas of section B in specimens of all groups showed a high degree of positive birefringence with loss of normal subsurface enamel structure (Fig. 2A). Notably, group I section A showed few demineralized areas intervening the normal subsurface enamel (Fig. 2B). However, group II section A revealed a dramatic decrease in normal enamel surface area with a noticeable positive birefringence, indicating an increased degree of demineralization. However, minimal areas of remineralized enamel were also noticed (Fig. 2C). Interestingly, group III section A disclosed almost typical homogenous subsurface enamel, portraying normal enamel mineralization and birefringence (Fig. 2D).

### Scanning electron microscopic results

**Fig. 3:**
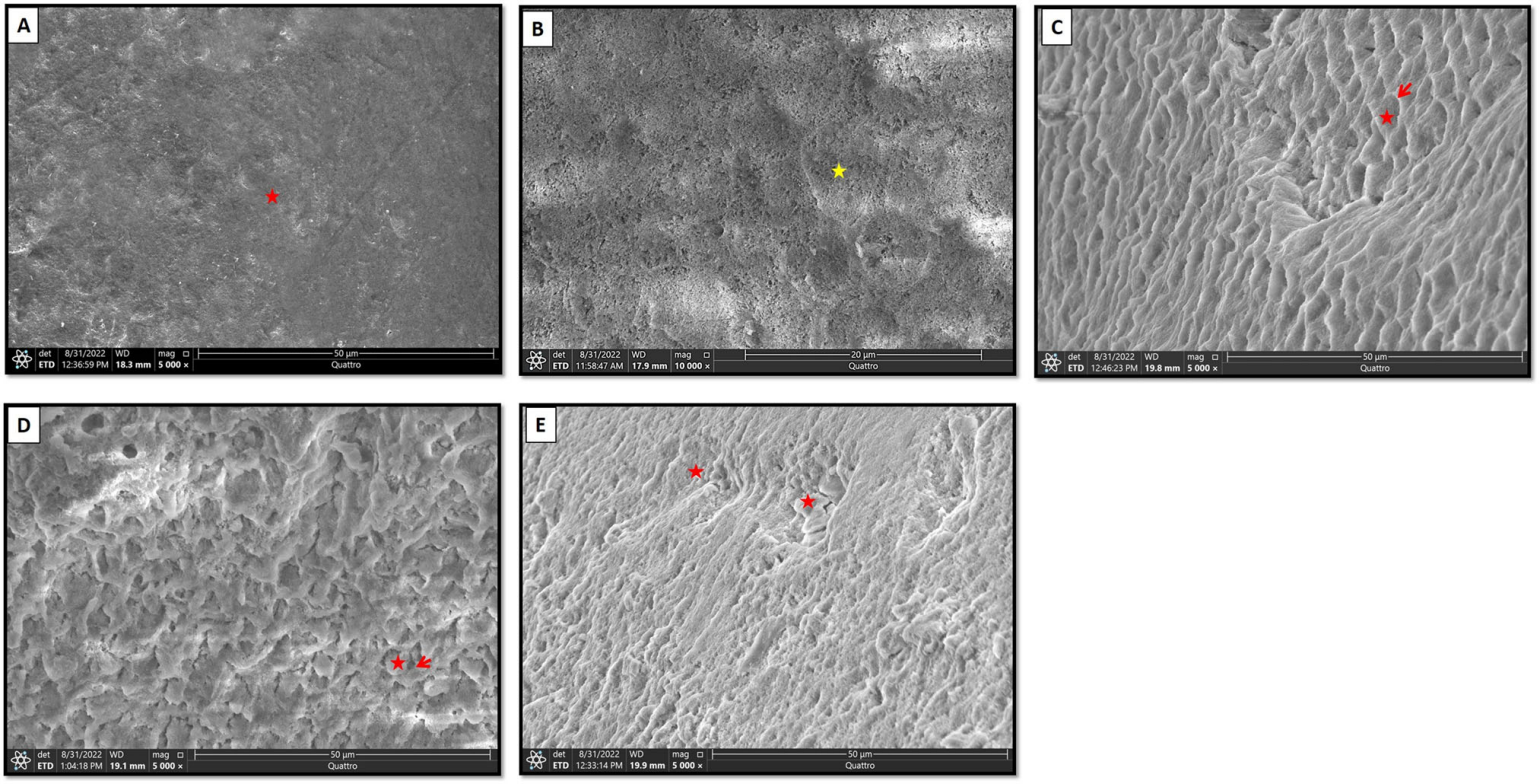
Scanning electron micrographs of normal enamel specimen and section B of all groups. **A** Showing smooth enamel architecture with homogenous aprismatic layer covering its surface (*asterisk*). **B** A higher magnification showing typical fish scale appearance (*asterisk*). **C** Showing type 1etching pattern with preferential removal of rod core (*asterisk*). *Arrow*; intact rod periphery. **D** Showing type II etching pattern where rod periphery was selectively removed (*arrow*). *Asterisk*; rod core. **E** Showing irregular etching pattern (*asterisk*). (SEM, original magnification; **A**, **C**–**E** × 5000, **B** × 20,000).

First of all, we had captured an SEM image for a typical, intact enamel surface to better comprehend the alterations occurred after treatments application. The intact specimen showed normal smooth enamel architecture with homogenous aprismatic layer covering the majority of its surface. On a higher magnification, some areas showed typical fish scale appearance of enamel rods (Fig. 3A, B).

Section B in specimens of all groups showed destruction of prismatic pattern. Irregular, rough and disorganized enamel surface was evident with noticeable increased porosities. Different types of etching patterns were detected including type 1, where enamel rod core was preferentially removed, and type II where the rod core remained relatively intact while its periphery was selectively removed. Also, an irregular etching pattern with pitted enamel surface was seen (Fig. 3C–E).

**Fig. 4:**
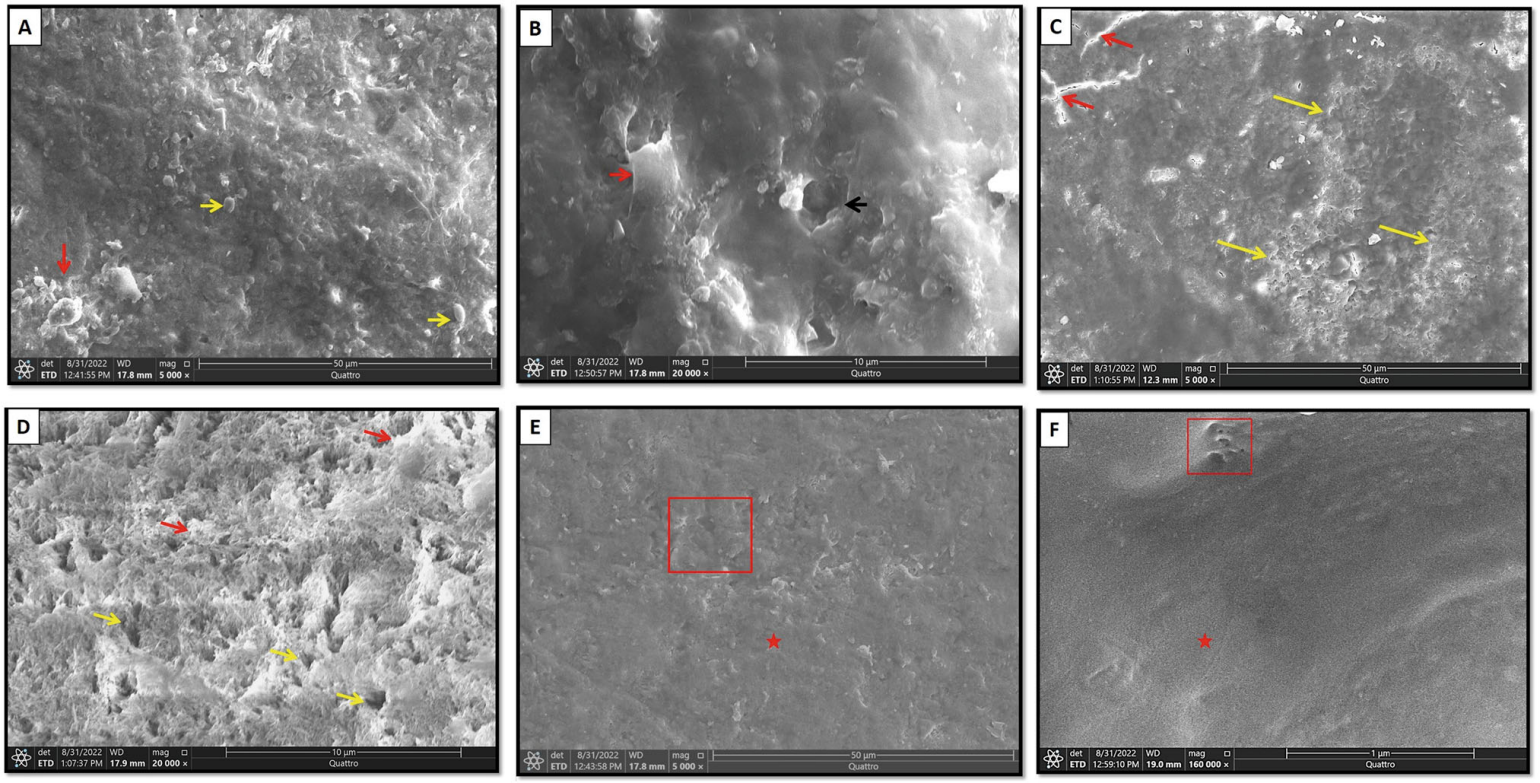
Scanning electron micrographs of group I section A, Group II section A and Group III section A. **A**, **B** Group I-A showing calcium deposits along the majority of enamel surface (*red arrows*) with detection of few micropores (*black arrow*). Residual NSF particles were adhered to enamel surface (*yellow arrows*). **C** Group II-A showing micro-cracks (*red arrows*) with detection of many scattered micro-pores (*yellow arrows*). **D** A higher magnification showing uneven enamel crystals with many porosities (*yellow arrows*) and minor amorphous surface precipitation (*red arrows*). **E** Group II-A showing homogenous enamel surface layer (*asterisk*). **F** A higher magnification of the boxed area of (**E**) showing minimal micro-pores (*boxed area*). *Asterisk*; homogenous enamel surface. (SEM, original magnification; **A**, **C**, **E** × 5000, **B**, **D**, **F** × 20,000).

In group I section A, globular precipitates were detected throughout enamel surface. Few porosities and defects of enamel had observed. Calcium deposits were revealed throughout enamel surface while the residual NSF particles were adhered to the enamel. Few micropores were also detected (Fig. 4A, B).

In group II section A, the enamel architecture was altered with almost total obscure of enamel rods. Some micro-cracks were shown along the major part of enamel surface with detection of many scattered micro-pores. A higher magnification showed uneven enamel crystals with many porosities and irregular topography with minor amorphous surface precipitation (Fig. 4C, D).

In group III section A, the enamel surface generally showed an overall even and homogenous architecture. The enamel rod structure was totally obscured by a homogenous surface layer. Minimal micro-pores were seen with higher magnification (Fig. 4E, F).

### Statistical analysis

#### EDX analysis of (Ca/P) ratio

EDX spectrometry was used to determine Ca and P weight % of the specimens. Calcium and phosphorus content were then converted into Ca/P ratios for each group. Concerning Ca/P ratio, (mean ± SD) of all specimens is shown in (Table 1A). The ratio in group I-A was higher than group I-B, and the difference was statistically significant (*P* < 0.05). On the other hand, when the mean Ca/P ratio values in group II-A and group II-B were compared, there was no recorded significant difference between the two values (*P* = 0.777). Interestingly, a highly significant increase in the mean Ca/P ratios were reported in group III-A compared to group III-B (*P* = 0.000**).

**Table 1:** Statistical analysis of the mean values of (Ca/P) ratio in group I, II and III.

**(A) Mean values of (Ca/P) ratio in group I, II and III**				
Groups	Mean ± S.D	Min --Max	*t*	*p* value
Group I-A	1.58 ± 0.28	1.2—1.9	4.345	0.002^a^
Group I-B	0.84 ± 0.26	0.5—1.1		
Group II-A	0.90 ± 0.21	0.7—1.2	0.293	0.777
Group II-B	0.86 ± 0.22	0.6—1.2		
Group III-A	2.06 ± 0.24	1.8—2.4	7.517	0.000^b^
Group III-B	0.92 ± 0.24	0.6—1.2		
*(B) Statistical analysis (ANOVA* *+* *Tukey test) between subgroup A of all groups*				
Groups	Mean ± S.D	Min --Max	*F*	*p* value
Group I-A	1.58 ± 0.28	1.2—1.9	28.311	0.000^b^
Group II-A	0.90 ± 0.21	0.7—1.2		
Group III-A	2.06 ± 0.24	1.8—2.4		
*(C) Groups*	*Group I*-*A*		*Group II-A*	
Group I-A	-------------		-------------	
Group II-A	0.002^a^		-------------	
Group III-A	0.023^a^		0.000^b^	

(A) Statistical analysis of the mean values of (Ca/P) ratio in group I, II and III for both section-A and section-B. (B) Statistical analysis of the mean values of (Ca/P) ratio in group I-A, II-A and III-A using ANOVA. (C) Pairwise Tukey-test of the mean values of (Ca/P) ratio in group I-A, II-A and III-A.

^a^Significant difference (*P* < 0.05).

^b^Highly significant difference (*P* < 0.001).

Remarkably, on comparing Ca/P ratios between all groups in section-A using ANOVA, a higher Ca/ P ratio was detected in group III-A (Table 1B). As, statistical analysis using pairwise Tukey-test (Table 1C), revealed a highly significant difference in the ratio in group III-A compared to group II-A. Also, there is a significant difference between group III-A and group I-A.

#### Shear bond strength measurements

The mean bracket bond strengths and standard deviations of subgroup A and B of all groups are presented in (Table 2A) When comparing subgroup A and B of each group, a highly significant difference was found between group I-A and B and also between group III-A and B. the difference was significant between group II-A and B (Table 2A*)*. According to one-way ANOVA, there was a significant difference among sub group A of all groups (Table 2B). Post hoc tukey test revealed significant difference between both group I-A and II-A and between group III-A and II-A. Although group III-A achieves the highest bond strength, the difference between it and group I-A was yet non-significant (Table 2C).

**Table 2:** Statistical analysis of the mean values of shear bond strength (MPa) in group I, II and III.

**(A) Mean values of Shear bond strength (MPa) in group I, II and III**				
Groups	Mean ± S.D	Min --Max	*t*	*p* value
Group I-A	152.81 ± 6.55	145.11—161.04	17.831	0.000^a^
Group I-B	79.38 ± 6.48	70.82—88		
Group II-A	98.49 ± 5.51	90.40—105	5.323	0.001^b^
Group II-B	82.46 ± 3.87	77.90—86.99		
Group III-A	182.08 ± 29.23	150—221	7.409	0.000^a^
Group III-B	82.91 ± 6.45	73--90		
*(B) Statistical analysis (ANOVA* *+* *Tukey test) between subgroup A of all groups*				
Groups	Mean ± S.D	Min--Max	*F*	*p* value
Group I-A	152.81 ± 6.55	145.11—161.04	29.098	0.000^a^
Group II-A	98.49 ± 5.51	90.40—105		
Group III-A	182.08 ± 29.23	150--221		
(C) Groups	Group I-A (NSF)		Group II-A(Diode)	
Group I-A	-------------		-------------	
Group II-A	0.001^b^		-------------	
Group III-A	0.053		0.001^b^	

(A) Statistical analysis of the mean values of shear bond strength in group I, II and III for both subgroup-A and B. (B) Statistical analysis of the mean values of shear bond strength in group I-A, II-A and III-A using one-way ANOVA. (C) Pairwise Tukey-test of the mean values of shear bond strength in group I-A, II-A and III-A.

^a^Highly significant difference (*P* < 0.001).

^b^Significant difference (*P* < 0.05).

## Discussion

Enamel is a unique epithelially-derived dental tissue, that possess inimitable microstructure and exceptional physico-chemical properties. Due to enamel unrestorable nature, many approaches were used to protect the tooth enamel against cariogenic challenge mainly white spot lesions development [9, 20, 27]. Many researchers used materials that are capable of controlling the development of caries, whether by triggering enamel remineralization or by inhibiting demineralization [28]. The latter approach aimed to reduce enamel mineral loss and lesion creation, thus reducing the solubility of enamel in acidic environments [29]. In order to evaluate the anti-cariogenic potential during the study, an artificial cariogenic challenge was conducted using combined use of demineralizing and remineralizing solutions during PH cycle. The later cycle was proved to simulate the onset and the progression of the carious lesion in vivo [24]. In addition, as orthodontic brackets cementation was proved to increase the possibility of plaque retention, enamel demineralization [30], brackets cementation was included in our study design.

The present in vitro study aimed to evaluate the effect of combined use of diode laser irradiation with nano silver fluoride varnish (NSF) on enhancing enamel anticariogenic activity around orthodontic brackets. PLM, SEM and EDX were utilized in the present study to monitor enamel resistance to cariogenic challenge. Further, brackets bond strength to enamel was evaluated after treatment.

Interestingly, this discussion will concentrate on two main queries. The first is what is the major causes for selecting the study materials. The second query does enamel surface pretreatment with these possibly anticariogenic approaches increase the enamel resistance to cariogenic challenge.

The first question is, what is the major causes for selecting NSF solely or combined with diode laser in the current study. NSF had been proposed to have preventive antimicrobial properties [31] and was portrayed to be an effective anti-caries agent [32]. Targino et al [20] also proved the antimicrobial and cytotoxic activity of NSF against streptococcus mutans. Because of the growing interest in the use of lasers in dental field, we combined NSF with diode laser. Recent researches had been used lasers as a new method of caries inhibition [33], others has been investigated lasers as a unique method used for the tooth surface modification and assisting its resistance to demineralization by acids [34]. On the molecular level, laser combination with a remineralizing agent was proved to cause microstructural changes in the irradiated dental hard tissue including melting and re-crystallization of areas exposed to the emitted laser light at suitable energy. These changes were proved to facilitate remineralizing agent particles absorption, thus, strengthening tooth structure against acid decalcification [16].

The second question is, does enamel surface pretreatment with these possibly anticariogenic approaches increase the enamel resistance to cariogenic challenge. The latter challenge was done in our study using PH cycling. This cycle was proved to simulate the dynamics of mineral loss and gain involved in caries development [35].

Regarding application time of NSF, we select 4 h to simulate the varnish application in dental clinics. As, after varnish application, patients are instructed not to rinse their mouth, not to drink or eat anything for 3 h, and not to brush till the next day [36, 37]. Thus, the actual application period was only 1 min and then we left it for 4 h before rinsing.

The sectioning model of each single tooth in each group into section A (test side) and section B (untreated side) was used in this study to gain the advantage that the two sides can be exposed to the same conditions with elimination of any possible bias between different teeth. Bracket bonding were applied to all specimens of all groups even those that were intended to be examined with SEM and PLM. This step was done to accurately mimic the natural oral environment in formation of WSLs around orthodontic brackets so, brackets were placed and the teeth were subjected to pH cycle. The brackets were later removed so that we can prepare ground sections and observe surface changes for both PLM and SEM respectively.

Upon viewing data obtained from the present study regarding the untreated section B in specimens of all groups, PLM showed a high degree of positive birefringence with loss of normal subsurface enamel structure. This was further assured by SEM analysis that showed development of initial enamel caries (white spot lesion) with destruction of prismatic pattern. As previously reported in many researches [38–40], different forms of enamel defects were observed including preferential removal of enamel rod core, preferential removal of rod periphery while rod core remained relatively intact and an irregular pitting of enamel surface [41, 42].

On the other hand, section A in all groups that received enamel surface pretreatment before PH cycling disclosed variable enamel response according to the treatment regimen. In group I section A, where enamel surface was treated with NSF prior to pH cycling, PLM showed few demineralized areas intervening the normal subsurface enamel. These results were in consistence with Nozari et al. [43] who portrayed that NSF had the greatest remineralization efficacy in primary teeth when compared to nanohydroxyapatite varnish. A closer look using SEM revealed calcium deposits sealing the enamel surface with globular precipitates were detected throughout enamel surface. The porosities and defects of enamel were remarkably fewer than that of section B. This came in accordance with Soekanto et al. [44] study where dentin discs, prepared from previously demineralized premolars, were pretreated with NSF then subjected to pH cycling. SEM analysis of their study showed formation of fluorapatite crystals with superior hardness and higher quality and intensity of apatite crystals. This can be explained by that the chief chemical reactions between silver fluoride compounds and hydroxyapatite include the creation of an impermeable layer of silver phosphate and calcium fluoride on the treated tooth surfaces [45].

Notably, group II section A, which received only diode laser irradiation prior to pH cycling, PLM revealed a dramatic decrease in normal enamel surface area with a noticeable positive birefringence. SEM viewed the enamel architecture to be altered with almost total obscure of enamel rods. Micro-cracks were shown along the major part of enamel surface with detection of many scattered micro-pores. A higher magnification showed uneven enamel crystals with many porosities and irregular topography with minor amorphous surface precipitation, indicating that only diode pretreatment could not prevent mineral loss. Similarly, a previous study of Aljdaimi et al. [46] emphasized the effect of laser irradiation upon tooth structure including enamel and dentin using nano-CT and SEM. In their study, the laser irradiated enamel surfaces displayed a distinctive rough morphology involving isolated cracks however with minute subsurface damage and preservation of the prismatic structure. Likely, they found a dense surface layer with flat glazed areas and they had referred it to the melting and subsequent cooling of enamel upon laser application.

Though, group III section A, that was treated with combined use of NSF and diode laser, displayed almost typical homogenous subsurface enamel, portraying normal enamel mineralization. The later result was in agreement with Hamoudi et al. [47] who concluded that the combined Nd:YAG laser illumination and silver nanoparticles decreased the tooth abrasion degree and enhanced tooth resistance to decay. They proposed that laser heating had a significant role in decreasing the stresses in hydroxyapatite lattice and aided in nanoparticles penetration to the laser-treated enamel surface, thus increasing the enamel resistance against acids. This was further confirmed by our SEM analysis which revealed the enamel surface to have an overall even and homogenous architecture with almost total preservation of typical enamel architecture

Interestingly, higher resistance against acids in group III section A was further proved by the observed higher Ca/P ratios in this group compared to section A in the remaining groups and section B in all groups. As, section B in all groups revealed a marked decrease in Ca/P ratio after immersion in the PH cycling solution, indicating enamel surface demineralization. Also, group II section A that was treated with laser only, recorded a decrease the Ca/P ratio. Although, samples of group I section A NSF-treated group have higher Ca/P ratios compared to the untreated section B in the same sample, indicating a low-rate of enamel surface decalcification. The later observation was in agreement with Zhi et al. [48] who found that that both silver and fluoride ions were responsible for enamel remineralization through the silver ions ability to infiltrate into carious lesions. Thus, combining laser irradiation with NSF in group III improved enamel resistance to cariogenic challenge.

Finally, statistical analysis of shear bond strength revealed a highly significant difference between group I-A and B and also between group III-A and B. The difference was significant between group II-A and B indicating that all the herein adopted anticariogenic techniques had a positive effect on increasing shear bond strength of the bracket to tooth surface. When comparing sub group A of all groups, the highest bond strength was detected in group III-A followed by group I-A. A significant difference was found between both group I-A and II-A and between group III-A and II-A, but the difference between group III-A and group I-A was non-significant. This came in accordance with the previously reported result of Favaro et al. [49] that dictated a statistically significant difference of shear bond strength between specimens treated with silver compounds, including silver diamine fluoride and nano silver fluoride, and untreated specimens. Also, Ergin et al. [50] reported that laser-activated bleaching system significantly enhances enamel shear bond strength when compared to other bleaching systems.

The limitations of this in vitro study were the inability to simulate oral environment regarding the salivary biofilm and oral flora, diverse salivary components together with different individual eating habits and oral hygiene practices. Also, the effect of diode laser treatment on the tooth dental pulp should be considered. Thus, further in vitro and in vivo research should be conducted to integrate the combined use of diode laser and NSF into dental restorative procedures to gain both antibacterial and remineralization properties. This unique combination should be used as an applicable product in adults and children with high caries incidence. Also, diode laser application at different powers should be assessed to determine its effect on the dental pulp temperature.

## Conclusion

The results of this study demonstrated that both anticariogenic agents, considered in this paper including laser radiation and NSF were found to affect dental enamel in relation to its surface morphology, chemical composition as well as bond characteristics. It can be concluded that the most superior enhancement in enamel criteria was achieved by surface pretreatment by combined NSF varnish and diode laser irradiation, which served to amazingly increase the acid-resistance ability of enamel. Considering the sole effect of NSF varnish and diode laser, NSF obviously provided a better anticariogenic effect for dental hard tissue. However, both diode laser and NSF evidenced to be promising treatment tools in the field of preventive dentistry to which we should refer to improve the efficacy of orthodontic management.

## Data Availability

All data of this study are available from the corresponding author upon request.
